# Role of task-based functional MRI in the assessment of sports-related concussion: a systematic review

**DOI:** 10.3389/fneur.2026.1720290

**Published:** 2026-03-13

**Authors:** Muhammad Mirza, Zubair Ahmed

**Affiliations:** 1 University Hospitals Birmingham NHS Foundation Trust, Birmingham, United Kingdom; 2 Department of Inflammation and Ageing, School of Infection, Inflammation and Ageing, College and Medicine and Health, Birmingham, United Kingdom; 3 Centre for Trauma Sciences Research, University of Birmingham, Birmingham, United Kingdom

**Keywords:** brain networks, concussion, fMRI, functional tasks, repeated head injury

## Abstract

**Introduction:**

Sport-related concussion (SRC) is a common and complex brain injury with variable recovery trajectories. The clinical assessment for SRC involves comprehensive assessment of symptoms and cognitive, visual and motor function, with tools such as the Sport Concussion Assessment Tool (SCAT) integrating these components for diagnosis and monitoring. Task-based functional magnetic resonance imaging (tb-fMRI) has emerged as a potential tool to evaluate functional brain changes post-concussion.

**Methods:**

A systematic review was conducted according to PRISMA guidelines. Five databases (Medline, Embase, PsycINFO, Scopus, Web of Science) were searched for original research studies up to July 2025 reporting tb-fMRI outcomes in SRC. Eligible studies included human participants with SRC undergoing tb-fMRI.

**Results:**

Of 1,130 records identified, plus 9 through manual searching, 15 studies met the inclusion/exclusion criteria for full text review, encompassing 273 SRC patients with an age range of 9–37 years old. A meta-analysis was not possible since numerical data was largely absent in studies. Across the studies, tb-fMRI revealed different patterns of altered brain activation, including both hypoactivation and hyperactivation during cognitive and sensorimotor tasks. Importantly, several studies showed altered activation persisted beyond symptom resolution suggesting functional recovery may lag behind clinical improvement.

**Conclusions:**

Task-based fMRI demonstrates consistent alterations in brain activity following SRC, particularly within frontoparietal networks. However, tb-fMRI findings were heterogeneous, sample sizes were small, and clinical applications such as return-to-play decisions based on functional imaging are not yet validated. To date tb-fMRI provides valuable insights into post-concussive brain function but remains an investigational tool and hence larger, standardized and longitudinal studies are needed to establish its clinical reproducibility and diagnostic utility.

## 1 Introduction

Sports-related concussion (SRC) involves a transient neurological impairment caused by biomechanical forces transmitted to the head (1) that lead to brain disturbances without necessarily producing detectable structural damage. Clinically, SRC is characterized by a broad and often subtle spectrum of physical, cognitive, emotional, and sleep-related symptoms, the variability of which complicates accurate diagnosis and recovery monitoring (2). A critical challenge in SRC management is that conventional structural imaging techniques, such as CT or MRI, typically appear normal despite ongoing dysfunction (3–5). By definition SRC remains a clinical diagnosis and is usually negative on conventional CT or MRI imaging (1). The presence of a radiological abnormality on standard clinical scans would generally reclassify the injury as a moderate traumatic brain injury rather than a concussion. However, the traditional severity-based TBI classification system is evolving toward a more mechanistic and spectrum-based framework, as discussed in recent literature (6). This diagnostic invisibility creates a gap in clinical care and return-to-play (RTP) decision-making, since there is often no objective marker of injury or recovery.

The transient and subjective nature of SRC symptoms frequently contributes to underreporting or misdiagnosis. Many athletes deliberately withhold symptoms to expedite RTP, while others fail to recognize subtle impairments (7). Such underreporting carries serious risks, including prolonged recovery, the development of persistent post-concussive symptoms, and increased vulnerability to repeat injury. In rare but severe cases, premature RTP may lead to Second Impact Syndrome, a potentially catastrophic outcome (8).

The diagnosis of SRC based on the recognition of characteristic symptoms and observable signs following a head impact, supported by standardized assessment tools such as SCAT is well established (1, 9). This approach however lacks objective, quantifiable biomarkers of injury or recovery, making it difficult to achieve definitive diagnosis or reliable prognostication (10, 11). Reliance on subjective measures further complicates the differentiation of SRC from other conditions or normal baseline variations in healthy individuals, highlighting the need for reliable biomarkers to detect subtle but important brain changes. This limitation has long been recognized and has prompted efforts to develop adjunctive diagnostic and prognostic models that integrate clinical findings with advanced imaging methods and, in some cases, serum biomarkers to improve diagnosis and prediction of recovery trajectories (12, 13).

Functional neuroimaging techniques particularly task-based functional magnetic resonance imaging (tb-fMRI) have gained attention as potential tools to bridge this gap. Task-based fMRI offers a non-invasive method for assessing brain function by measuring blood-oxygen-level-dependent (BOLD) activity during specific cognitive tasks (14). This approach can detect differences in activation patterns following concussion, even when structural imaging appears normal (14, 15). By examining the brain's response under cognitive load, task-based fMRI provides insight into altered neural processing after concussion, which may ultimately help guide patient management and RTP decisions.

In tb-fMRI paradigms, participants typically alternate between task performance and control or rest conditions, enabling comparisons of task-evoked activation patterns. Commonly used tasks include working memory, attention, and inhibitory control paradigms, as these reliably engage neural circuits vulnerable to SRC (16). Across studies, SRC has been associated with both hypoactivation and hyperactivation relative to controls, suggesting disrupted and compensatory neural responses rather than a uniform pattern of dysfunction (14).

Although the BOLD signal represents an indirect measure of neuronal activity, reflecting vascular and metabolic coupling rather than direct neuronal firing, it provides valuable insight into functional brain dynamics under task demand (17–19). By linking altered brain activation to specific cognitive processes, tb-fMRI could shift SRC assessment from subjective symptom reports to objective biomarkers (20). The aim of this systematic review was to evaluate the diagnostic utility, limitations, and future potential of tb-fMRI in the context of SRC.

## 2 Materials and methods

### 2.1 Protocol and reporting

The systematic review was conducted in accordance with the Preferred Reporting Items for Systematic Reviews and Meta-Analyses (PRISMA) 2020 guidelines (21). The review protocol was developed *a priori* and followed standard methodology for evidence synthesis in neuroimaging research.

### 2.2 Data sources and search strategy

A comprehensive literature search was performed across five electronic databases: Medline, Embase, PsycINFO, Scopus, and Web of Science up to 16 July 2025, with no date restrictions. Search terms combined keywords (MeSH and Boolean terms) and controlled vocabulary related to sports-related concussion (SRC), mild traumatic brain injury (mTBI) and task-based functional MRI (tb-fMRI). Reference lists of included studies and relevant reviews were manually searched to identify additional eligible studies. Only studies involving SRC were included to ensure homogeneity of mechanism, demographic characteristics, and clinical context. Civilian and military mTBI studies were excluded due to differing injury mechanisms, comorbidities, and recovery profiles, which could confound interpretation of sport-specific neurofunctional findings. The search was conducted by M.M. and confirmed by Z.A., using the same terms (Table 1).

**Table 1:** Showing databases search terms used and any limits applied to the literature review search.

**Search string used for electronic database searches**	
Head injury and sports	exp Post-Concussion Syndrome/or exp Brain Concussion/or concussion.mp. (mTBI or mild traumatic brain injury) craniocerebral trauma.mp. or exp Craniocerebral Trauma/Head Injuries, Closed/or blunt head trauma.mp. acquired brain injury.mp. traumatic brain injury.mp. or exp Brain Injuries, Traumatic/or exp Traumatic Brain Injury/or exp Athletic Injuries/ Youth Sports/or Sports/or Racquet Sports/or Water Sports/or Snow Sports/or sports.mp. sport^*^.mp. (recreation or recreat^*^ or baseball or bicycling or boxing or cycling or diving or equestrian or equine or football or “Head Protective Devices” or helmet^*^ or hockey or lacrosse or “martial arts” or karate or judo or “tae kwon do” or aikido or mountaineering or “racquet sports” or rugby or skating or skiing or snow sports or soccer or wrestling).mp. exp ball sports athlete/or exp athlete/or exp contact athlete/or exp student athlete/exp Australian football/or exp football/or exp gaelic football/or exp football player/exp Sports/or exp “Athletic and Sports Personnel”/or exp High School Sports/or exp Adaptive Sports/or exp Extreme Sports/or exp College Sports/or exp Professional Sports/(sportsmen or sportswomen).mp.
fMRI	fMRI.mp. or exp Magnetic Resonance Imaging/resting state fMRI.mp. rs-fMRI.mp. task based fMRI.mp. blood oxygen level dependent fMRI.mp. BOLD fMRI.mp. functional connectivity.mp. functional magnetic resonance imaging.mp. blood oxygen level dependent imaging.mp. functional neuroimaging.mp. or exp Functional Neuroimaging/

### 2.3 Eligibility criteria

Studies were included if they met the following criteria: Population: human participants with a diagnosis of SRC or sports-related mTBI. Intervention/Exposure: use of task-based fMRI to assess brain function. Outcomes: reported neural activation findings (e.g., BOLD signal changes) during cognitive, motor, or sensorimotor tasks. Study type: original peer-reviewed research articles. Although our inclusion criteria focused on diagnosed SRC, a small number of studies examining repetitive sub-concussive head impacts were considered when their design, participant population, and imaging paradigms closely paralleled those used in concussion research. These were included to provide contextual insight into cumulative neural effects of repetitive head trauma but were analyzed and interpreted separately from acute concussion studies.

Exclusion criteria were; case reports, case series, reviews, editorials, meta-analyses, animal studies, studies employing imaging modalities other than tb-fMRI (e.g., resting-state fMRI, DTI, MRS, PET), multimodal neuroimaging studies where the contribution of tb-fMRI could not be isolated, and studies not specific to SRC (e.g., general mTBI without a sports-related mechanism, combat trauma).

### 2.4 Study selection

All identified records were imported into a reference manager, and duplicates were removed. Two reviewers (M.M. and Z.A.) independently screened titles and abstracts for relevance. Full texts of potentially eligible articles were retrieved and assessed in detail against the inclusion and exclusion criteria. Since there were no disagreements during the study selection process, discussion between the two primary authors nor consultation with a third reviewer was needed to reach consensus.

### 2.5 Data extraction and synthesis

Data were extracted using a standardized template, including study design, participant characteristics (sample size, age, sex, sport), concussion diagnosis criteria, time since injury, task paradigms, imaging parameters, and main tb-fMRI outcomes [regions of hyperactivation or hypoactivation]. Given methodological heterogeneity across studies, and a lack of numerical data, a qualitative synthesis of findings was performed, and a meta-analysis was not possible.

### 2.6 Risk of bias and quality assessment

The methodological quality of included studies was assessed, independently by both authors (M.M. and Z.A.) using the JBI (Joanna Briggs Institute 2020) Checklist for Cohort Studies tool (22). The JBI critical appraisal tool is a standardized checklist used to evaluate the methodological quality of studies and to determine the extent to which they have minimized or excluded systematic errors. The results indicate that most of the included studies have a moderate risk of bias, with 11 studies falling into this category and only four studies deemed to have a low risk of bias (Table 2).

**Table 2:** Characteristics of the studies.

**References**	**Study origin**	**Study design**	**No. of participants**	**Age**	**Gender M/F**	**Level of play**	**Sport**	**JBI risk of bias**
Jantzen et al., 2004 (28)	USA	Prospective cohort	8 (4 SRC, 4 controls)	20 years; 19–23 years	M	NCAA football	Football	Moderate
Chen et al., 2004 (31)	Canada	Prospective cohort	24 (16 SRC, 8 controls)	Control: 26.9 ± 5.2 years; Concussed: 26.9 ± 7.2 years	M	Mixed competitive athletes	Mixed	Moderate
Chen et al., 2007 (23)	Canada	Prospective cohort	28 SRC (18 PCS) + 10 controls	Low PCS: 26.9 ± 5.6 years; moderate PCS: 30.8 ± 5.8 years; control: 21.9 ± 1.6 years	M	Competitive athletes	Mixed	Moderate
Chen et al., 2008 (32)	Canada	Prospective cohort	15 (9 SRC + 6 controls)	Control: 20.0 ± 0.9 years; SRC-PCS Improved Group: 33.8 ± 5.6; SRC PCS Not Improved Group: 29.6 ± 5.1 years	M	Competitive sports	Mixed	Low
Chen et al., 2008 (24)	Canada	Prospective cohort	40 SRC, 16 Controls	Control: 20 ± 1.2 years; SRC no depression: 26 ± 5.6 years; SRC mild depression: 29 ± 6.7 years; SRC moderate depression: 30 ± 7.4 years	M	Competitive athletes	Mixed	Moderate
Lovell et al., 2007 (34)	USA	Prospective cohort	28 SRC + 13 controls	16.6 ± 2.4; 13–24 years	M/F	HS/college	Mixed	Moderate
Pardini et al., 2010 (25)	USA	Prospective cohort	16 SRC	16.3 years, 14–23 years	M/F	Collegiate	Mixed	Low
Dettwiler et al., 2014 (33)	USA	Prospective cohort	15 SRC + 15 controls	19.8 ± 0.9 years	M/F	Varsity athletes	Mixed	Low
Talavage et al., 2014 (29)	USA	Prospective cohort	11 SRC + sub-concussive group	15–19 years (mean 17.0 years)	M	HS football	Football	Moderate
Keightley et al., 2014 (27)	Canada	Prospective cohort	15 SRC + 15 controls	Control: 14 ± 2.3 years; concussed: 14.5 ± 2.3 years	M/F	Youth sports	Mixed	Moderate
Johnson et al., 2015 (35)	USA	Prospective cohort	9 SRC+, 9 Controls-	20–22 years	M/F	Collegiate	Mixed	Moderate
Slobounov et al., 2010 (36)	USA	Prospective cohort	15 SRC + 15 controls	Control: 21.3 years; concussed: 20.8 years	M/F	Collegiate	Mixed	Moderate
Hammeke et al., 2013 (30)	USA	Prospective cohort	12 SRC, 12 Controls	Control: 16.5 ± 0.52 years; concussed: 16.5 ± 0.52 years	M	Collegiate	Football	Low
Sinopoli et al., 2014 (37)	Canada	Prospective cohort	13 SRC, 14 Controls	Control: 12.6 ± 1.6 years; concussed: 12.6 ± 1.6 years; 9–15 years	M	Youth	Mixed	Moderate
Breedlove et al., 2012 (26)	USA	Prospective cohort	24 Season 1; 28 season 2	Season 1: 17.0 years, 15–18 years; Season 2: 16.8 years, 14–18 years	M	High school	Football	Moderate

The appraisal involved detailed examination of each study's methodology, including whether the concussed and control groups were comparable at baseline, whether the exposure and outcomes were measured in a valid and reliable manner, and whether potential confounding factors were identified and addressed. We also considered the sufficiency of the follow-up period and whether incomplete follow-up was handled appropriately. Across the included studies, overall methodological quality was moderate. Most studies met fundamental design and reporting standards but showed limitations in sample size and control of confounding variables. While nearly all studies matched participants by age and sex, few accounted for additional factors such as time since injury, number of previous concussions, or baseline cognitive performance. Only a small number explicitly reported statistical adjustment for these confounders linking BOLD activation with symptom severity, behavioral performance, or head-impact metrics (23–27). Technical parameters including scanner vendor, field strength, and preprocessing steps were inconsistently reported, reducing reproducibility and comparability across datasets.

As a result, risk-of-bias assessments indicated moderate concerns in most studies, primarily due to incomplete confounder control and inconsistent methodological transparency. Overall, while the existing body of evidence provides valuable insights into post-concussion brain function, the reliability of these findings is constrained by methodological variability and moderate study quality (Table 2).

## 3 Results

**Figure 1:**
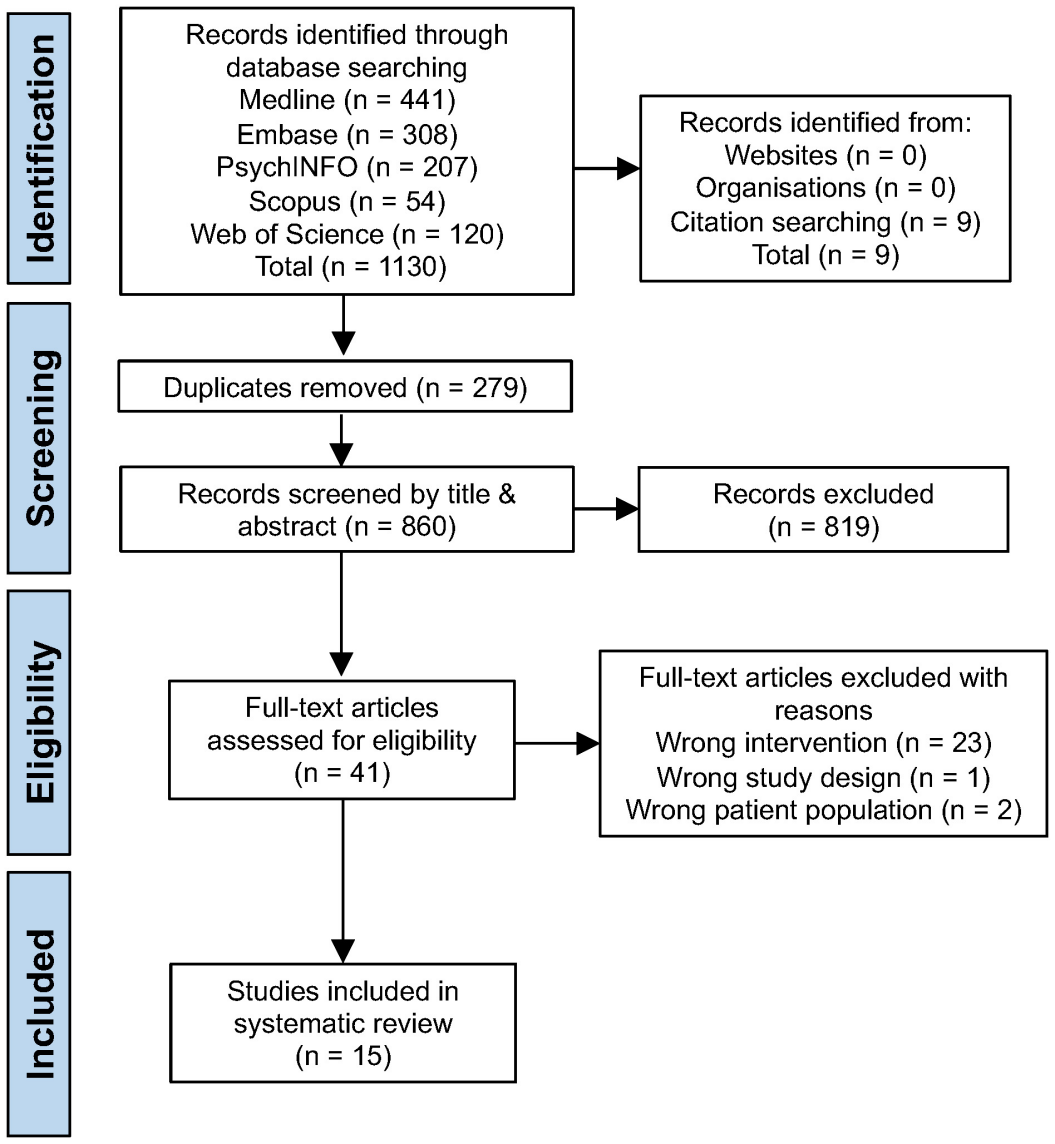
PRISMA flow chart.

The database search identified 1,130 studies and an additional 9 studies were located through manual citation searching, yielding a total of 1,139 studies. After duplicates were removed 860 studies remained. Following title and abstract screening, 41 studies were assessed in full-text for eligibility. Ultimately, 15 prospective cohort fMRI studies met all inclusion and exclusion criteria and were included in this systematic review (Figure 1).

### 3.1 Characteristics of the studies

Nine of the included studies originated from US and six from Canada (Table 2). All studies were a prospective cohort design and included male and female participants aged of 9 to 37 years comprising a total of 236 SRC and 152 control participants. One study did not report the exact distribution of participants between concussed and control groups (26). The athletes represented a range of competition levels, from professional to collegiate and high school sports. Four studies (26, 28–30) focused exclusively on football players whilst the remaining studies investigated mixed sport cohorts (23–25, 27, 31–37). Participants were scanned across a range of recovery phases, acutely (within 72 h−2 weeks post injury), sub-acutely (~2–6 weeks), and longitudinally (up to 2 months post injury). Several studies included both symptomatic and asymptomatic athletes and/or youth samples. Four of the 15 studies were judged to have a low risk of bias (25, 30, 32, 33) while 11 studies had moderate risk of bias (23, 24, 26–29, 31, 34–37) (Table 1).

Most studies employed working-memory (WM) paradigms (12/15), while single studies used oculomotor/anti-saccade, dual-task, or sensorimotor paradigms (1/15 each) (Table 3). Across all studies, altered recruitment of the frontoparietal control network was consistently observed (15/15), with occasional additional visual/cerebellar involvement (1/15). Behaviourally, two-thirds of studies reported no or only minor group differences in task performance despite robust BOLD differences (10/15), whereas just over half also documented measurable impairments such as slower or less accurate performance, or oculomotor errors on the administered task or related neurocognitive tests (8/15). These categories were not mutually exclusive (Table 3).

**Table 3:** Outcomes of behavioral tests.

**References**	**Behavioral test**	**Behavioral outcomes**
Jantzen et al., 2004 (28)	Mathematical, memory and sensorimotor coordination tasks (finger sequencing, serial calculation, digit span tasks).	No behavioral differences post-concussion. Performance in digit span and calculation not changed between baseline and post-concussion sessions.
Chen et al., 2004 (31)	Verbal and visual working memory tasks (externally ordered).	Poor performance on subtraction task in concussed group. No performance accuracy differences vs controls, although concussed athletes seemed to have more difficulty with the visual task.
Chen et al., 2007 (23)	N-back working memory task (2-back vs. 0-back).	Neurocognitive impairments early; recovery over time. Concussed athletes demonstrated significantly worse performance on IMPACT at initial assessment but improved to control levels on recovery.
Chen et al., 2008 (32)	Verbal and nonverbal working memory (externally ordered-pseudo-words vs. control task).	Moderate PCS group: slower response times than controls on 1-back and matching tasks. Low PCS group response accuracy and speed comparable to controls on both versions of WM task
Chen et al., 2008 (24)	Externally ordered working memory (pseudo-words vs. control task).	No differences in response accuracy or speed between groups or between the two study periods.
Lovell et al., 2007 (34)	Externally ordered working memory task (pseudo-words vs. control task).	No significant differences in response accuracy or speed between any of the groups. But there was a trend of less accurate and lower performance in concussed athletes with depression
Pardini et al., 2010 (25)	Virtual reality memory navigation tasks (encoding, retrieval, random navigation, active navigation).	No differences in performance between groups in terms of task success rate and time to completion.
Dettwiler et al., 2014 (33)	N-back working memory task (0-back vs. 2-back).	No difference in performance accuracy; Symptom severity was not significantly correlated with N-back performance indicating that more symptomatic individuals did not have significantly more difficulty accurately completing the N-back task
Talavage et al., 2014 (29)	N-back working memory task (2-back vs. 1-back).	Verbal working memory deficits were predominate in COI+/FOI+ individuals, while COI-/FOI+ individuals tended to exhibit stronger deficits in visual working memory
Keightley et al., 2014 (27)	Sternberg working memory task (Event related-Encode, maintenance, response phases).	Acute: At 13 h post-injury, concussed athletes were less accurate and had slower reaction times and greater inter-trial variability on Sternberg task accuracy. By 7 weeks, no significant differences were observed between the groups.
Johnson et al., 2015 (35)	N-back working memory task (verbal/spatial working memory task for *n* = 1, 2, 3).	No differences in performance accuracy or reaction time between concussed and control groups at any of the three time points.
Slobounov et al., 2010 (36)	N-back visual working memory task.	The COI +/FOI + group exhibits an onset and extent of behavioral deficits consistent with damage to integrative centers of the brain associated with auditory (especially language) processing. The COI-/FOI+ group predominantly exhibits behavioral deficits in working memory (predominantly visual).
Hammeke et al., 2013 (30)	N-back working memory single (visuospatial) vs dual-task (N-back + motor task).	Dual task: concussed group vs controls, slower responses, accuracy maintained suggesting a speed accuracy trade-off. Not observed in the single task condition.
Sinopoli et al., 2014 (37)	Verbal and nonverbal working memory tasks (externally ordered).	Concussed youths performed significantly worse across all accuracy conditions of the working memory tasks with no difference in reaction time. Also significantly poorer performance on the baseline condition of each task.
Breedlove et al., 2012 (26)	A battery of 7 Oculomotor tasks with simultaneous eye tracking (saccade, smooth pursuit, anti-saccade etc.).	Concussed subjects performed poorer than controls, showed longer latencies, lower accuracy, higher error rates, increased position errors, and fewer self-paced saccades on the more demanding tasks (anti-saccades, self-paced saccades, and memory guided saccades).

ROI, Region of Interest; COI, Clinically observed impairment; FOI, Functionally observed impairment.

Across the 15 studies, eight reported predominantly hyperactivation, five reported predominantly hypoactivation, and two reported mixed patterns (Table 4). Hyperactivation refers to greater BOLD signal intensity and/or a larger spatial extent of suprathreshold activation in concussed participants compared with controls, whereas hypoactivation refers to reduced BOLD signal intensity and/or smaller activation. One study additionally reported attenuated task-negative deactivation, and another described longitudinal normalization of activation over time. Some studies contributed to multiple categories depending on the timepoint examined, which accounted for overlap between classifications (Table 4).

**Table 4:** Outcomes from fMRI studies.

**References**	**Timing of fMRI**	**fMRI outcome measures**	**Direction of fMRI effect**
Jantzen et al., 2004 (28)	~1 week post-SRC, 1 end of season	BOLD signal amplitude and extent change in ROIs (within-subject change vs baseline)	**Hyperactivation**—Marked increases in amplitude and extent of BOLD activity post-concussion. Concussed players had marked increases in amplitude and extent of BOLD activity in bilateral inferior-superior parietal, dorsolateral and frontal, and cerebellar regions particularly during the finger-sequencing task.
Chen et al., 2004 (31)	1–14 months (mean 4.7)	BOLD signal amplitude and spatial extent in ROIs (within-subject change vs baseline)	**Hypoactivation**—Reduced or absent activation in symptomatic athletes; normalization after recovery. Symptomatic athletes had significantly weaker BOLD changes in the right mid-dorsolateral prefrontal cortex compared to controls, even with similar performance, showed additional activations in posterior cerebral regions including temporal and parietal lobes.
Chen et al., 2007 (23)	5 ± 6.4 months post SRC	Activation magnitude and extent during WM tasks, linked to ImPACT outcomes	**Hyperactivation**—Hyperactivation early during the first week predicted clinical recovery. Athletes with greater hyperactivation in a midline frontal network took significantly longer to return to play (45.8 days). Lower activation in posterior parietal network was associated with higher symptom severity.
Chen et al., 2008 (32)	Study 1: 1–9 months post-SRC; Study 2: 12 ± 7 months after study 1	Task-related BOLD signal changes vs PCS scores	**Hypoactivation**—Reduced task-related activation in DLPFC; abnormal peaks outside ROI. The moderate PCS group had slower cognitive performance and reduced fMRI signals in DLPFC. Both concussed groups showed additional peak in the left temporal lobe not seen in controls.
Chen et al., 2008 (24)	4.9–7.3 months depending on group	BOLD activation levels in WM circuits; longitudinal changes with recovery	**Mixed**—Study 1: atypical activation acutely in right DLPFC and left Temporal lobe in PCS Improved group after concussion with no activation in left DLPFC. With symptom resolution in Study 2 increased activation in left DLPFC but no activation of Right DLPFC & Left Temporal Lobe.; PCS Not Improved group showed atypical activation in left premotor cortex, dorsal anterior cingulate cortex and bilateral rostral insula but NO activation in left DLPFC in either study 1 or 2.
Lovell et al., 2007 (34)	6.6 ± 4.7 days and 33.3 ± 33.8 days post SRC	BOLD activation magnitude correlated with depression symptom severity	**Hypoactivation**—Concussed athletes with depression symptoms showed reduced activation in the DLPFC and striatum and attenuated deactivation in rostral anterior cingulate cortex, posterior cingulate cortex, medial orbitofrontal cortex, and bilateral parahippocampal gyrus. This attenuation was positively correlated with severity of depression, the reduction of neural activity in these areas appears to be inversely correlated with activity in DLPFC.
Pardini et al., 2010 (25)	3–12 days postinjury; median: 6.5 days post-SRC	Cluster size, BOLD percent change during encoding vs retrieval	**Hyperactivation**—Asymptomatic concussed athletes showed, larger cluster size and greater BOLD percent signal change during encoding in right DLPFC, parietal cortex, and right hippocampus. No significant difference between groups during retrieval.
Dettwiler et al., 2014 (33)	≤ 2 days, 2 weeks and 2 months post-SRC	BOLD activation magnitude correlated with PCS symptom severity	**Hyperactivation**—Greater symptom severity is related to hyperactivation and compensatory recruitment. There are circumscribed bilateral frontal and parietal cortical regions where increased activation is correlated with symptom severity. The areas of activation in more highly symptomatic concussed individuals occur near the borders of activated areas in less symptomatic concussed individuals, indicating recruitment of additional cognitive resources during the acute concussed phase.
Talavage et al., 2014 (29)	Preseason, in-season (within 48 hrs of event) and post-season (1–3 months after event)	BOLD activation changes across 116 ROIs; regression vs head impact telemetry	**Mixed**—Neurophysiological changes correlated with cumulative head impacts. Regressions found significant relationships between the number and location of blows and neurophysiological changes for both concussed (COI+/FOI+) and asymptomatic groups with functional impairment (COI-/FOI+). The starkest difference is the substantial involvement of upper parietal and occipital visual processing systems in the COI-/FOI+ group contrasted with the involvement of upper temporal and lower parietal verbal processing systems in COI+/FO+ group.
Keightley et al., 2014 (27)	9–90 days post-SRC	Load-dependent BOLD activation; longitudinal change with recovery	**Hypoactivation/Hyperactivation**—Decreased activation acutely; compensatory increased activation at 7 weeks. During the acute phase (13 h), injured athletes showed under activation of right hemisphere attentional network during all three task phases (encode, maintenance, response). At 7 weeks, this pattern reversed, with injured athletes showing compensatory hyperactivation in the same networks. The opposite pattern with injured subjects having greater activation in Right Insula and lower activation at 7 weeks relative to controls.
Johnson et al., 2015 (35)	Within 7 days post-SRC	Percent BOLD signal change in WM networks, longitudinal (3 time points)	**Hyperactivation**—Concussed athletes showed persistent, significantly increased activation in the bilateral DLPFC in all three sessions, persisting up to 2 months despite normal behavioral performance and symptom resolution. Increase activation in left inferior parietal lobe also noted persisting up to 2 weeks.
Slobounov et al., 2010 (36)	≤ 30 days post-SRC	BOLD WM task activation and signal percent change	**Hyperactivation**—Altered activation in DLPFC even without clinical symptoms in COl-/FOI+ group, who showed abnormal ImPACT scores and decreased fMRI activation in the DLPFC and cerebellum. This was linked to a high number of head collision events. The COI+/FOI+ group had significant altered activation in left middle and superior temporal gyri.
Hammeke et al., 2013 (30)	13 h and 7 weeks post-SRC	BOLD activation magnitude in WM and dual-task networks	**Hyperactivation**—No activation of working memory network in the 1-back task, instead activity in left midfrontal and lingual gyrus, with 2-back condition concussed group recruits working memory components including right DLPFC, right inferior parietal, right premotor and left precuneus region. With dual task activation of left DLPFC, other aspects of prefrontal cortex, posterior parietal cortex, caudate and cerebellum. No further activation of left DLPFC beyond the 1 back dual task condition.
Sinopoli et al., 2014 (37)	3–6 months post-SRC	Task-related BOLD activation correlated with WM accuracy	**Hypoactivation**—Reduced activation in concussed vs controls. Concussed youths showed significantly reduced activity in bilateral DLPFC, left premotor cortex, supplementary motor area, and left superior parietal lobule during working memory tasks. Additionally reduced activation in dorsal anterior cingulate cortex, left thalamus, left caudate nucleus during nonverbal task.
Breedlove et al., 2012 (26)	Preseason and in-season follow-up for concussed athletes	BOLD activation during oculomotor control tasks + eye tracking performance	**Hyperactivation**—Recruitment of additional brain regions, larger activation clusters in concussed athletes. The concussed group showed widespread hyperactivation in the cerebellum, frontal, parietal, temporal, and occipital lobes, DLPFC, Visual Cortex, left frontal eye fields precuneus and brainstem compared to the control group in response to oculomotor tasks.

Working memory and executive function paradigms revealed both hypoactivation and hyperactivation in prefrontal and parietal cortices depending on injury stage. Multiple studies reported reduced dorsolateral prefrontal cortex (DLPFC) activation in symptomatic athletes compared with controls (24, 31, 32, 34, 36, 37), while others demonstrated compensatory hyperactivation within frontal and parietal networks, including right DLPFC, superior parietal lobule, and supplementary motor area, despite preserved task accuracy (23, 25, 26, 28–30, 33, 35). Longitudinal designs showed that these activation differences persisted beyond clinical symptom resolution, with atypical patterns still observed up to 2 months post-injury (23, 25, 30). These findings indicate disrupted executive network efficiency, where hypoactivation reflects reduced engagement and hyperactivation compensatory effort. Their persistence beyond symptom resolution suggests neural recovery lags behind clinical recovery. None of the reviewed studies demonstrated a validated statistical association between tb-fMRI activation patterns and clinical recovery trajectory or RTP outcomes. Although several longitudinal investigations described partial or complete normalization of activation concurrent with symptom improvement, these relationships were observational rather than predictive. Variability in sample size, timing of assessments, and analysis methods prevented firm conclusions about how fMRI-based changes relate to recovery progression.

Spatial navigation and oculomotor paradigms also demonstrated persistent alterations. In asymptomatic athletes recently cleared to return to play, virtual reality navigation tasks elicited enlarged recruitment of right hippocampus, parietal cortex, and prefrontal areas compared with controls (36). Oculomotor tasks in acutely concussed athletes revealed altered activation within visual–cerebellar–oculomotor circuits, associated with longer latencies, increased anti-saccade errors, and reduced self-paced saccade generation (24). Follow-up testing in the subacute phase showed partial improvement but persistent over-recruitment in motion-sensitive visual areas and cerebellum (24). These results suggest lingering disruption of visuospatial and oculomotor control networks after concussion. Persistent over-recruitment of hippocampal, parietal, and cerebellar regions likely reflects compensatory mechanisms to maintain performance, indicating that functional recovery continues beyond symptom resolution.

Symptom burden was consistently linked to neural activation. Higher post-concussion symptom scores were associated with broader or inefficient cortical recruitment and reduced DLPFC activation during working memory tasks (18, 21, 28, 31). Although several studies incorporated clinical symptom measures such as the Post-Concussion Symptom Scale (PCSS) or the Rivermead Post-Concussion Symptoms Questionnaire (RPQ) to quantify ongoing symptom burden, only a subset, examined direct correlations between symptom severity and fMRI activation patterns (24, 25, 27, 30, 32, 33, 35). In general, greater symptom severity particularly in cognitive and emotional domains was associated with increased recruitment of prefrontal and parietal regions, suggesting compensatory hyperactivation, whereas individuals reporting predominantly physical or fatigue-related symptoms sometimes showed reduced activation, consistent with diminished neural efficiency. The limited number of studies performing such analyses, combined with variability in symptom assessment tools and timing of evaluation, restricts firm conclusions. Athletes with persisting post-concussive symptoms also demonstrated abnormal prefrontal and temporal activation patterns, including associations with depressive symptomatology (24). Youth athletes demonstrated reduced working memory accuracy alongside diminished activity in bilateral DLPFC, anterior cingulate, thalamus, and caudate, indicating a more limited capacity for compensatory recruitment compared with adults (27, 29).

Functional changes were also reported in athletes without a clinically diagnosed concussion, where altered DLPFC recruitment and modest visual working-memory differences were observed (29). However, these findings were derived from a very small subsample and relied on the ImPACT test, which has limited test–retest reliability and low diagnostic precision. Similarly, another study (26) found that cumulative and location-specific head-impact exposure among high-school football players correlated with widespread fMRI alterations despite the absence of diagnosed concussion, supporting the hypothesis of cumulative neural strain. These sub-concussive studies were not combined with acute SRC data but were analyzed and interpreted separately to provide contextual insight into the broader spectrum of repetitive head trauma. Their findings were considered exploratory and descriptive, illustrating possible cumulative effects rather than serving as evidence of acute concussion pathophysiology. Accordingly, interpretations related to recovery or clinical outcome were restricted to studies involving clinically confirmed SRC, and no claims regarding diagnosis or RTP determination were inferred from tb-fMRI results.

Evidence of sub-concussive effects further supported this continuum. High school football players without clinically diagnosed concussion but with frequent head impacts exhibited measurable neurophysiological impairments, including altered DLPFC recruitment and visual working memory deficits (26, 29). Study examining cumulative and location-specific impacts (26) reinforced the possibility of dose-dependent neural effects from repetitive head trauma. Collectively, these findings highlight that greater cognitive or emotional symptom burden is often associated with compensatory hyperactivation in prefrontal and parietal regions, whereas physical or fatigue-related symptoms can correspond with reduced neural efficiency. These findings highlight a dose–response relationship between symptom burden and neural activation after concussion. Although limited by heterogeneous symptom measures and small sample sizes, the overall evidence suggests that persistent symptoms and repetitive head-impact exposure may contribute to prolonged or cumulative disruption of executive and visuospatial networks.

### 3.2 Brain networks activated in tb-fMRI

Task-based fMRI consistently revealed frontoparietal control network as the most reliable signature of sports-related concussion (SRC) (Table 4). Alterations in the dorsolateral prefrontal cortex (DLPFC) and parietal regions were reported in all paradigms, with both hypo- and hyperactivation patterns observed depending on clinical stage and symptom burden. Hyperactivation was commonly observed in symptomatic or subacute athletes (25), consistent with compensatory recruitment of additional neural resources. In contrast, hypoactivation was more frequently reported in youth (27) and in athletes with persistent post-concussive symptoms (23, 31), reflecting reduced neural efficiency and diminished capacity for compensation.

The cerebellar network was prominently engaged in acute oculomotor tasks, where concussed athletes displayed widespread hyperactivation across cerebellum, brainstem, and motion-sensitive visual cortices, alongside increased anti-saccade errors and longer latencies (35). Cerebellar involvement was also observed during high load working memory and attentional tasks (26). These findings suggest that additional cerebellar and oculomotor resources are recruited to maintain performance under acute disruption, supporting the use of oculomotor paradigms as sensitive biomarkers of early injury. Persistent symptoms, particularly those linked to affective burden, were associated with altered activation in limbic and emotional networks. Athletes with depressive symptomatology exhibited reduced activity in DLPFC and striatal regions alongside attenuated deactivation in medial prefrontal and temporal cortices (24). These findings indicate that depression after concussion reflects underlying pathophysiology in limbic–frontal circuits, which may contribute to greater disability and poorer outcomes.

Subcortical networks including thalamus, caudate, and striatum were implicated in several studies of persisting symptoms (23, 24) and in youth cohorts (27). Differences in activation in these regions highlights disruption of cortico-striato-thalamic loops critical for cognitive control. In youths, this was accompanied by reduced working memory accuracy and diminished bilateral DLPFC, anterior cingulate, and caudate activation (27), suggesting limited ability to engage compensatory pathways compared with adults. The visual and hippocampal networks also showed consistent alterations. Spatial navigation paradigms revealed enlarged recruitment of hippocampus, DLPFC, and parietal cortex in recently concussed but asymptomatic athletes (36). Functional changes were also evident in athletes with no diagnosed concussion, where altered DLPFC recruitment and impaired visual working memory performance were observed (29). Similarly, cumulative and location-specific head impacts correlated with widespread fMRI changes across frontal, temporal, cerebellar, and visual cortices (26). These results demonstrate that fMRI can detect hidden disruption in navigation and visual–cognitive circuits, even when behavioral measures appear normal.

Overall, the evidence suggests several key points. First, the frontoparietal network is the core substrate of concussion-related dysfunction, with compensatory hyperactivation emerging as the most frequent pattern of alteration (23, 25, 27, 31). Second, cerebellar, limbic, subcortical, and hippocampal networks contribute variably depending on task domain, symptom profile, and age (24, 26, 29, 35, 36). Third, differences in activation patterns frequently persist beyond clinical recovery, with longitudinal evidence showing continued prefrontal hyperactivation up to 2 months post-injury (33). Finally, fMRI reveals differences in activation between symptomatic and asymptomatic athletes; however, none of the included studies demonstrated a validated association between these activation patterns and recovery duration, symptom burden, or RTP outcomes (23–25, 27, 29, 31, 33, 35, 37). Consequently, tb-fMRI should be regarded as an adjunct that provides complementary insights into neural function rather than an established clinical biomarker of recovery or severity.

## 4 Discussion

In this systematic review we aimed to evaluate the diagnostic utility, limitations, and future potential of tb-fMRI in the context of SRC. From a total of 1139 articles, 15 met our inclusion/exclusion. Across the studies, tb-fMRI revealed heterogeneous patterns of altered brain activation, including both hypoactivation and hyperactivation during cognitive and sensorimotor tasks. Notably several studies demonstrated that altered activation persisted beyond symptom resolution, raising concerns regarding RTP decisions and susceptibility to repeat injury (25, 28, 33, 35, 36). Consistent variations in brain activity were observed following sport-related concussion, particularly within frontoparietal networks. However, findings were heterogeneous, sample sizes were small and clinical applications such as return-to-play decisions have not yet been validated (38). Of the 15 studies, 11 were rated as moderate quality, limiting the strength of the conclusions and underscoring the need for larger high quality studies to confirm these observations.

Interpretation of altered activation in tb-fMRI requires contextualization within models of cognitive processing. Approximately half of the included studies (23, 25, 27, 30–32, 35, 36) discussed their findings within frameworks such as compensatory recruitment, neural inefficiency, or resource-demand models. In these studies, hyperactivation was typically interpreted as increased neural effort or compensatory engagement of additional cortical regions to maintain task performance, while hypoactivation reflected reduced network efficiency or limited resource availability under cognitive load. The remaining studies primarily reported activation differences descriptively without embedding them within a formal cognitive or computational model. Consequently, increases or decreases in BOLD signal should not be regarded as inherently positive or negative, rather, they represent different modes of neural adaptation that must be interpreted in the context of the cognitive task and recovery stage.

Exploratory evidence from studies of repetitive head impacts (26, 29) suggests that cumulative exposure may produce functional alterations even without a clinically diagnosed concussion. Recent neuroimaging research supports this continuum, showing subtle white-matter and functional network alterations with repetitive head-impact exposure, even in the absence of overt clinical concussion (39, 40). These studies broaden the discussion of neural vulnerability in contact sports but must be interpreted with caution. Very small sample sizes, and reliance on neurocognitive instruments with limited reliability such as ImPACT reduces confidence in the reported associations. Consequently, while these findings raise important hypotheses about sub-concussive effects and cumulative neural strain, they remain preliminary. Although sub-concussive exposure was not the primary focus of this review, its inclusion in a limited number of studies (26, 29) offers insight into the broader continuum of neural vulnerability associated with repetitive head impacts. These findings were interpreted separately from those of acute concussion and should be viewed as exploratory and hypothesis-generating, given their small sample sizes, limited control for impact quantification, and absence of clinical diagnosis.

Building on this broader context, tb-fMRI provides important insights into the pathophysiology of SRC. While concussion is clinically defined as a transient neurological disturbance, fMRI demonstrates that alterations in brain function may persist well beyond symptom resolution. These changes typically manifest as variable recruitment of frontoparietal and subcortical systems during working memory, executive, memory, visuospatial, and oculomotor tasks, indicating continuing inefficiency or compensatory adaptation within cognitive control circuits. Collectively these findings support several key interpretations of post-concussive brain function and its clinical relevance, as detailed below.

### 4.1 Beyond behavioral measures

Changes in brain activation patterns detected by fMRI often persist long after an athlete's symptoms have resolved and performance on neuropsychological tests has normalized. Longitudinal studies show persistent dorsolateral prefrontal cortex (DLPFC) and parietal hyperactivation despite clinical recovery (33). Similarly, athletes clinically cleared for return to play demonstrated enlarged recruitment of the hippocampus and DLPFC regions during spatial navigational tasks, despite preserved task accuracy (36). Athletes without clinically diagnosed concussion also exhibited altered DLPFC activation and visual working-memory deficits (29), while cumulative and location-specific sub-concussive impacts correlated with widespread fMRI alterations (26). Symptom-linked hyperactivation in working memory tasks with preserved accuracy further highlights this gap between clinical and physiological recovery (25). These findings indicate that neural recovery may lag behind symptom resolution, suggesting persistent physiological disruption even after clinical normalization and emphasizing the need for objective measures beyond symptom reports to assess true recovery.

The spatial distribution and clinical correlates of activation changes highlight fMRI's potential as a candidate biomarker of concussion severity and prognosis. Symptom burden consistently predicted inefficient cortical recruitment and reduced DLPFC activation (23, 25). In athletes with persistent post-concussive symptoms, depressive symptomatology was linked to reduced DLPFC and striatal activation, alongside attenuated deactivation in medial temporal regions (24). Serial imaging revealed dynamic patterns of fMRI changes, with hyperactivation normalizing in parallel with symptom resolution (33). Youth athletes demonstrated reduced working-memory accuracy and hypoactivation of bilateral DLPFC, thalamus, caudate and anterior cingulate cortex, indicating limited capacity for compensation compared with adults (27). Sub-concussive exposure further reinforced the cumulative impact, as blow counts and impact location predicted neural alterations (26). Collectively, these findings suggest that tb-fMRI may capture physiological recovery trajectories, with prefrontal inefficiency reflecting greater symptom burden and normalization paralleling clinical improvement. Developmental and exposure-related differences, however, underscore the complexity of applying fMRI as a prognostic tool.

### 4.2 Implications for RTP

Persistent differences in brain activation patterns suggest that athletes may face heightened risk of re-injury if RTP decisions rely solely on symptom resolution. Evidence of residual prefrontal and parietal abnormalities beyond clinical recovery (33), as well as enlarged hippocampal, parietal, and prefrontal recruitment during virtual reality (VR) navigation in asymptomatic athletes (36), indicates delayed physiological recovery. Similarly, high-school players without diagnosed concussion showed measurable neurophysiological changes (29). Together these findings suggest that neurophysiological recovery may drop behind clinical recovery supporting the integration of objective physiological measures alongside symptom-based assessments in RTP protocols.

However, despite these observations, none of the included studies demonstrated a statistically validated relationship between tb-fMRI activation patterns and clinical recovery trajectory or time to RTP. Reported changes such as partial normalization of hyperactivation were descriptive rather than predictive and derived from small, heterogeneous cohorts. Sample sizes, follow-up intervals, and analytical approaches were not sufficiently robust to establish a definitive relationship between fMRI-derived measures and recovery duration or completeness. Consequently, while tb-fMRI highlights ongoing physiological processes after clinical recovery, its role in predicting recovery or informing RTP decisions remains unproven and requires further longitudinal validation.

The utility of neuropsychological and symptom-based assessments in RTP decision-making also remains debated. Computerized neurocognitive tools such as ImPACT show limited test–retest reliability and diagnostic precision, while the Sport Concussion Assessment Tool (SCAT) is optimized for acute identification within the first 24–72 h and lacks validation for clearance decisions (41, 42). RTP determinations should therefore not rely on these tools or on imaging findings alone. Instead, an evidence based, multimodal approach integrating clinical examination, graded exertional testing, vestibular-ocular and balance assessments, and longitudinal symptom monitoring remains the current best practice. Within this framework, tb-fMRI may serve as a potential adjunct in concussion assessment, providing insight into physiological recovery dynamics that are not captured by clinical or cognitive testing. However, its clinical adoption requires further validation, methodological standardization, and demonstration of prognostic reliability before it can be incorporated into RTP decision-making.

### 4.3 Brain strain: the role of compensation

Many studies document hyperactivation and atypical recruitment patterns, consistent with compensatory neural mechanisms. Symptom-linked hyperactivation in DLPFC and parietal regions during working memory tasks despite preserved accuracy (25) as well as enlarged recruitment during spatial navigation tasks, despite normal behavior (36) reflect compensatory effort. In the acute phase, oculomotor tasks elicited widespread hyperactivation in frontal eye fields, motion-sensitive visual cortex, cerebellum, and brainstem, accompanied by longer latencies and increased anti-saccade errors (35). Dual-task studies in youth athletes revealed slower performance and atypical frontoparietal recruitment, consistent with inefficient cortical allocation under divided attention (37). Importantly, when compensatory mechanisms failed as in youth athletes with reduced accuracy and bilateral DLPFC hypoactivation, vulnerability to functional impairment became apparent (27). Overall, these findings suggest that hyperactivation reflects compensatory neural effort to preserve performance following concussion, whereas reduced capacity for such recruitment, particularly in younger athletes, indicates diminished neural efficiency and greater susceptibility to functional deficits.

Normative studies establish baseline activation trajectories for comparison with SRC. In healthy participants, increasing working memory load produces linear increases in frontoparietal, supplementary motor, insular, and cerebellar activation with increasing task difficulty, alongside deactivation of default mode regions (43). Connectivity within working memory networks also scales with task load (44). In contrast, concussed athletes often deviate from these patterns, exhibiting exaggerated activation at low task demands or premature plateauing at moderate loads. Such deviations confirm that concussion disrupts both the efficiency and adaptability of neural recruitment.

The mechanisms underlying these fMRI changes can be understood through the convergence of several pathophysiological models. The concept of neural inefficiency is consistent with the neurometabolic cascade that follows concussion, wherein ionic flux, glutamatergic excitation, mitochondrial dysfunction, and altered cerebral blood flow produce an energy crisis (11, 45). Task-fMRI - hyperactivation may therefore reflect increased cortical effort required to maintain function under reduced energetic reserves. At the same time, structural models emphasize the role of diffuse axonal injury and network disconnection, where shearing of white matter tracts disrupts connectivity and slows information transfer across distributed systems (46–48). The resulting inefficiency manifests as hyperactivation at low task demand, inability to scale at higher demand, or hypoactivation in chronic states (25, 31, 33). Network-level models further suggest that, inefficient switching between task-positive and task-negative states undermines flexible engagement of control networks, leading to greater reliance on frontal over-recruitment (49, 50).

Both developmental and sex-related factors appear to modulate these neural responses. Females often exhibit distinct activation patterns and recovery trajectories compared with males suggesting that hormonal and structural factors may influence both vulnerability and efficiency of neural repair (51, 52). Children and adolescents with immature prefrontal circuitry are less able to engage compensatory hyperactivation and more prone to performance decline (27, 53). Beyond neurobiological determinants, psychological and contextual moderators including depression, stress, and fatigue have also been linked to altered activation patterns and impaired neural efficiency (23–25, 54). These findings underscore the multifactorial nature of concussion, where physiological, developmental, and psychosocial factors interact. Finally, persistent inefficiency observed in otherwise asymptomatic athletes may represent an early functional marker of chronic neuroinflammation or neurodegeneration. Processes such as microglial activation and abnormal tau accumulation, driven by repetitive head impacts, have been implicated in the pathogenesis of chronic traumatic encephalopathy (11, 55–57). Tb-fMRI may thus capture subtle functional signatures of ongoing neurobiological processes before clinical deficits emerge.

These converging models suggest that tb-fMRI changes arise from multiple overlapping mechanisms involving metabolic vulnerability, axonal disconnection, network dysregulation, cerebellar involvement, developmental limitations, sex differences, and chronic inflammation. This complexity helps explain the variability in recovery trajectories and highlights why subjective symptom reporting alone is insufficient to fully capture physiological recovery. Despite its potential, significant challenges remain before fMRI can be clinically integrated as a tool into the diagnosis and prognosis of SRC. Methodological variability continues to limit reproducibility across studies, making it difficult to clearly define its role in SRC. Differences in task design, duration, and cognitive load, along with variations in MRI acquisition parameters such as scanner vendor (Siemens, Philips, or GE) and magnetic field strength (1.5T, 3T or 7T) as well as differences in preprocessing pipelines and analysis software (e.g. FSL, SPM, AFNI, CONN) all contribute to inconsistent results (23, 27, 37, 58–60). Heterogeneity in control group selection and incomplete reporting of baseline characteristics, such as age and sex further limit comparability, while small sample sizes restrict statistical power and generalisability (27, 37, 53). Consequently, some discrepancies in reported results likely reflect methodological inconsistencies rather than genuine neurobiological variation (60, 61). Feasibility is further constrained by the high cost and limited availability of MRI, as well as the limited availability of expertise required for data acquisition, processing and interpretation of results (9, 11, 21, 33). Even with high-quality data, substantial inter-individual variability and the lack of robust baseline datasets stratified by age, sex, and athletic level complicate interpretation (62). At present, the clinical value of fMRI should be regarded as adjunctive, offering complimentary insights alongside established assessment tools. It may help clarify uncertain diagnoses, support RTP decisions when symptoms have resolved but neural inefficiencies persist, and guide individualized rehabilitation strategies targeting specific network-level alteration (24, 35, 36). In summary, this systematic review demonstrates that task-based fMRI reveals consistent yet heterogeneous alterations in brain activation following SRC, particularly within frontoparietal and subcortical networks. These functional changes often outlast symptom recovery, reflecting persistent neural inefficiency or compensatory adaptation. While the collective evidence underscores fMRI's potential to enhance diagnostic and prognostic precision, methodological heterogeneity, limited sample sizes, and inconsistent confounder control limit current clinical translation. Standardized, high-quality studies integrating behavioral, physiological, and imaging data are required to validate tb-fMRI as a reliable biomarker of concussion recovery and to inform safe, individualized RTP decisions.

### 4.4 Limitations

This review has several important limitations. The included studies were small (often enrolling fewer than 30 participants) and heterogeneous with respect to imaging tasks, timing of assessment, and analytic approaches. Scanner parameters and preprocessing methods were inconsistently reported, limiting reproducibility and comparability across cohorts. Methodological variability, including differences in task design, cognitive load, scanner provider, magnetic field strength, and data acquisition, likely contributed to the diversity of findings observed across studies. Risk-of-bias assessments indicated moderate concerns in most studies reflecting incomplete reporting, limited blinding, and variability in participant selection and confounder control.

Interpretation of tb-fMRI findings as evidence of underlying neural dysfunction should be approached with caution. In the context of task-based paradigms, activation differences between concussed and control groups are only meaningful when accompanied by demonstrable and clinically significant behavioral impairments on the same task. Most studies did not include such behavioral validation or report discriminative metrics to substantiate claims of dysfunction. Consequently, observed differences in activation should be regarded as descriptive of altered neural processing rather than definitive indicators of impaired function or recovery.

Our search strategy was limited to English-language publications, and gray literature was not systematically searched: therefore, relevant studies may have been missed. Additionally, although some included studies involved athletes exposed to repetitive head impacts without diagnosed concussion, these were interpreted separately from acute SRC studies. Their inclusion was intended to provide contextual insight into cumulative neural effects but may have reduced the specificity of conclusions. Although focusing exclusively on SRC limited the number of included studies, this restriction was necessary to maintain population and task homogeneity. Broader inclusion of civilian mTBI or military cohorts, while increasing sample size, would have introduced substantial variability in injury mechanism, comorbidity and recovery patterns, thereby limiting applicability to athletic populations.

Feasibility and translation remain constrained by high cost, limited access, and the specialized expertise needed for data acquisition and interpretation, which together limit the immediate clinical utility of fMRI.

## 5 Conclusion

Task-based fMRI consistently demonstrates alterations in brain activity following sport-related concussion, most prominently within the frontoparietal control network. However, the direction, spatial extent and magnitude of these changes vary across tasks and stages of recovery, and the small, heterogeneous evidence base precludes firm conclusions about their clinical significance. Based on the current evidence, tb-fMRI should be regarded as an investigational tool that enhances understanding of the neurobiology of concussion rather than a validated biomarker for diagnosis, prognosis or RTP decision-making. Integration with complementary modalities including structural MRI/DTI and blood-based biomarkers has begun to clarify the physiological basis of recovery, with studies demonstrating linked trajectories between diffusion metrics, fMRI measures, and peripheral biomarkers in both concussed and head-impact–exposed athletes (63). These multimodal associations support the premise that tb-fMRI could evolve into a clinically actionable adjunct if validated in larger, standardized cohorts.

## Data Availability

The original contributions presented in the study are included in the article/supplementary material, further inquiries can be directed to the corresponding authors.
